# Human-centered AI through employee participation

**DOI:** 10.3389/frai.2024.1272102

**Published:** 2024-03-01

**Authors:** Thomas Haipeter, Manfred Wannöffel, Jan-Torge Daus, Sandra Schaffarczik

**Affiliations:** ^1^Faculty of Social Sciences, Institute for Work, Skills and Training, University of Duisburg-Essen, Duisburg, North Rhine-Westphalia, Germany; ^2^Department of Social Sciences, Ruhr-University Bochum, Bochum, Germany; ^3^Gemeinsame Arbeitsstelle RUB/IGM, Bochum, Germany

**Keywords:** human-centered AI, employee participation, works council, ethic rules, company agreement on AI

## Abstract

This article examines the role of employee participation in AI implementation, focusing on a case study from the German telecommunications sector. Theoretical discussions highlight concepts of employee participation and workplace democracy, emphasizing the normative basis for human-centered AI in Europe. The empirical analysis of the case study demonstrates social practices of human-centered AI and the importance of employee representatives and labor policies in sustainable technology. The contribution is structured into two main parts: first, discussing sociological concepts of employee participation and summarizing the role of works councils in shaping digital technology implementation. Second, focusing on a case study of AI regulations at Deutsche Telekom, highlighting the significant effects of employee participation and co-determination by the group works council in promoting socially sustainable AI implementation which is done via qualitative case analysis. The article highlights the significance of participation and negotiations and gives an example for social partnership relations in AI implementations.

## Introduction

This article is about the role of employee participation in the process of AI implementation both from a theoretical and an empirical point of view by looking at a case study of the telecommunication sector from Germany. On the one hand theoretical outlines give emphasis to some concepts of employee participation and workplace democracy for specifying the normative basis of human-centered AI at work in the European context. On the other hand, the case study analysis presents social practices of human-centered AI to specify criteria of the role of employee representatives and labor policy to implement digital technologies in a sustainable way. In coordinated market economies like the German one management strategies and the implementation of new technologies are strongly shaped by social institutions and regulations of labor relations. However, the way this process of shaping works and the following effects are not determined by the mere existence of social institutions themselves, but by concrete strategies and activities of the actors of labor relations and by the power resources and capabilities these actors can rely on.

The contribution is structured in two steps. Firstly, we will discuss some sociological concepts of employee participation like participation, labor process analysis or production models that can be used for the analysis of employee participation in AI implementation. In this context we will also summarize what is already known about the ways works councils do actively shape the implementation of digital technologies during the last years (Haipeter and Schilling, 2023; Hirsch-Kreinsen, 2023a,b; Kuhlmann, 2023; Pfeiffer, 2023).

Secondly, we will focus on an internationally broadly discussed and recognized practice case of the AI-regulations of “Deutsche Telekom,” in which employee participation and the co-determination of the group works council proved to produce rather important effects for a social sustainable implementation of AI, developing three instruments: a Manifesto, a digital roadmap and new form of agile IT company agreements (Bargmann, 2022; Doellgast et al., 2022; Doellgast, 2023; Doellgast and Kämpf, in press). At “Deutsche Telekom,” since 2016 the group works council and management have agreed on several company agreements concerning the introduction of digital technologies and especially on the implementation and the use of AI.^1^ They have developed an “AI Manifesto” which takes in account the general ethical guidelines of the AI Act of the European Commission and the national AI-Strategy of the German Government.^2^ The “AI-Manifesto” intents to structure decision-making processes about the introduction of new AI-systems with new forms of agile company agreements. These new agreements give the works councils an important say in the process of application including a veto-right, and it includes principles that have to be met by new IT-systems. Taking these instruments together, the new forms of agile company agreements and the “AI-Manifesto” represent a particularly far-reaching form of participation of works councils and employees in the telecommunication sector. The following analysis is about potential challenges for employees' participation in the process of the implementation of AI.

The issue of employee participation in the context of AI implementation illuminates the relationship between social institutions and economic practices by focusing on the agency of institutionalized actors. The analysis explores the conditions and activities which allow institutionalized actors to become effective in shaping digital transformations. Effectivity here means both, the fact that the implementation of technologies is influenced by these social actors and that labor policy at company level is an important instrument to protect employment and working conditions. This seems to be even more important as there is an ongoing political debate in the EU and within EU member states about the further development of the EU AI Act and the improvement of legal opportunities for information, consultation and participation.

## Key concepts of employee participation

This chapter presents some concepts of employee participation and argues that these concepts have an analytical surplus value for understanding the role and the forms of employee participation might have in the implementation of AI or other forms of digital technologies. The first of these concepts is *participation* itself, which is traditionally among the key concepts of labor and industrial sociology. In former times, participation has been regarded as a quality of collective action of trade unions or other representations of employee interests. In this sense it was regarded as part of “industrial citizenship rights” of employees (Marshall, 1950). In any case, in this view collective action took place beyond the limits of the individual organization of companies: either like in the British tradition of “industrial democracy,” as a quality of collective bargaining between employers and—independent as well as professionalized—trade unions; or like in the German tradition of “economic democracy,” as a result of trade union participation in the centralized planning of the economy. There was no independent role of direct participation on company or establishment level given in these overarching concepts (Haipeter, 2019a).

However, and on the contrary to this, nowadays participation is recognized as an independent element of labor relations *besides* collective bargaining. Whereas the latter is about collective contracting of labor standards, the former is about having a say in the concrete conditions under which labor is used in the organization of the labor process. This means that direct participation is the cornerstone of what can be called “democracy at work,” based on certain status rights workers can dispose of beyond the contractual conditions of the sale of labor power, be they legally and/or collectively agreed (Dukes and Streeck, 2023).

Participation as an analytical concept of its own emerged during the 1960s, driven both by the fact that in several European countries statutory rights of participation on establishment and company level have been implemented in course of the postwar reconstruction of the economies and driven by the critique of the representative structures of the labor movements that developed during the 1960s. From then on, participation has been regarded as a democratic element within the economy that is based on influencing firms' decision-making both in a representative way by labor representatives and in a direct way by employees themselves within establishments and companies. As such, participation has become an important concept in comparative research about industrial relations and at the same time an interdisciplinary concept also used in organization or HR theory (Wilkinson, 2011).

Moreover, participation in this sense can rest on very different forms, ranging from information to consultation and to codetermination. In the case of information, workers or their representatives have to be informed about managerial decisions; consultation means that they are able to articulate their interest about these decisions which can then be included or ignored in the decision-making process; and in the case of codetermination, finally, the decision cannot be made without the consent of the workers. In most of the European countries with statutory participation rights, these rights refer only to information and consultation (Haipeter, 2019a). This is also true for the European level and its core institution of European Works Councils. One of the most important exceptions from this rule is Germany, where the statutory rights of participation also include codetermination, at least with respect to certain topics of the implementation of new technologies.

The German case is instructive for our analysis, both because it includes the most developed forms of participation in the sense of codetermination and because we refer to it in our case study of the role of the group works council of “Deutsche Telekom.” Codetermination rights, as they are listed mainly in the renowned § 87 of the German Works Constitutions Act, extend over several issues, ranging from the distribution and position of working times working times to wage methods or the organization of teamwork. Of special importance for digitalization issues are the § 87.6, which ensures codetermination in case of the introduction and application of technical instruments that might be used to monitor the conduct and performance of employees, and the § 87.14, which is about codetermination on the regulation of mobile work which is based on IT-technologies. Furthermore, the § 80.3 which was adopted in the Works Council Modernization Act of 2021 nowadays gives the works councils the opportunity to consult an external expert in case AI systems are introduced and have to be assessed by the works councils without any permission by the management.

Participation in this sense can be regarded as a bundle of institutionalized collective status rights of employees. However, as legal norms these rights tell us little about how labor policy does function in concrete social situations and in how far they actually shape social practices. Here a second important concept to deal with employee participation comes into play, the notion of the *labor process* as a terrain of politics, conflicts and contests. This view has been developed in the British Labor Process Debate, which stresses the aspect of control in managerial strategies as a means to regulate and monitor the labor process and to cope with the problem of transforming labor power into concrete labor (Thompson, 1990).

However, as this research tradition has shown rather different strategies of control can be distinguished. Control does not mean that management tries to monitor every aspect of the labor process. Instead, control strategies may range between the extreme poles of direct control—like in Taylorist forms of scientific management with high levels of division of work, rigid separation of execution and control and the concentration of the knowledge of the labor process in the hands of management—on the one and responsible autonomy on the other hand, the latter giving the workers broad leeway to apply their qualifications and knowledge (Friedmann, 1977). In this perspective, management not only has choices to make, but there is also room for contestations, negotiations, and compromises between management and labor about control issues at work.

In a complementary way, Edwards (1979) has distinguished three forms of control as an expression of the “structured antagonism” that characterizes the relationship between labor and capital on the shopfloor which is constantly negotiated and re-negotiated. Control in this sense means a system of political regulation. The three forms of control systems according to Edwards are, first simple or personal control by managers and superiors supervising the labor process, technical control by the demands and connections of technological artifacts and machines like the assembly line, and, finally, bureaucratic control by the institutionalization of control in the form of job descriptions or rules of promotion. The two latter forms depersonalize and, in this way, according to Edwards, mystify the control relationships as independent technological necessities or institutional rules.

This analysis connects control and conflicts about control issues with the aspect of consent as a precondition for stable control systems. According to Burawoy (1985), work contexts are characterized by three strongly connected dimensions: the economic dimension of the production of things or services, the political dimension in the sense of the production of social relations, and the ideological dimension by producing experiences of those relations. Interests of workers and management are coordinated within the political and ideological dimensions of work on the shop floor, producing a hegemony within more or less stable work regimes which are not continually contested. This mostly applies to regimes based on a more or less stable balance of power between labor and capital, much less however to coercive or despotic hegemonial regimes in which power and coercion are visible and may lead to contention.

Given these understandings of the labor process, labor process theory suggests to analyse the digitalization of the labor process with respect to issues like the skilling or deskilling of labor, the effects on the autonomy and responsibility of the workers, the control regimes and the ways digital technologies contribute to or modify existing control strategies and, finally, to the production of consent about the implementation of digital technologies in the labor process (also Briken et al., 2017).

However, as Thompson and Laaser (2021) argue, looking at technology it makes sense to distinguish first and second order strategies of management, with first order strategies concerning the development and adoption of technological systems in interactions between firms, state actors and scientific-professional domains, whereas second order strategies are about the implementation of technologies and concrete strategies of control and about negotiations and contestations of these strategies. Furthermore, in line with the concept of *production models* which connects the dimensions of company strategies including finance and product strategies, process organization including the labor process, and labor relations between management and labor representatives (Boyer and Freyssenet, 2003), the authors argue that the control regime is embedded in a regulatory regime of labor regulation and an accumulation regime including conditions of competition and finance.

As research has shown, employee participation and the way it can be implemented in conflicts about autonomy, control or qualifications largely depends on the *power resources* the actors can rely on in the labor process (Schmalz and Dörre, 2014). The most important of these resources for an effective employee participation are: (1) structural power, which is based on market and organizational positions of employees and which gives them either individual power or power for the collective actors in which they are organized; (2) organizational power in terms of high trade unions density or the ability to mobilize workers in concrete conflicts related to issues of participation; and (3), institutional power, which is based on the legal rights of employee representation in companies, both in terms of the organization of these actors and the concrete rights of information, consultation or more advanced forms of participation they can dispose of. It has been stressed in literature that in the context of digitalization a fourth form of power may play an important role, which is discursive power which shapes the way digital technologies are interpreted, either as instruments of autonomy and improvements of working conditions or as instruments of competitiveness, rationalization and control (Kuhlmann and Rüb, 2020).

However, this analysis is about potential topics and issues without looking at the concrete agency of labor representatives and workers and the conflicts, negotiations or new forms of consent that might develop around these issues. This kind of analysis needs in-depth case studies also in combination with industry studies in order to better understand business policies on digitalization and the role of labor relations and regulations the implementation is embedded in. This is what this article tries to show for the case of the role of the group works councils of the Deutsche Telekom. Before we do this, we will give a short overview on the findings concerning digitalization and the role of codetermination and trade unions in Germany.

## Works councils and employee participation in digitalisation processes

What do we know about the role of works councils and trade unions in German play in digitalization processes? Do they participate actively in these processes, do they shape conflicts and consent in the labor process, and do they influence the development of production models? The findings on this question are quite ambiguous at first glance (Kuhlmann, 2023). This is especially true with regard to works councils, which as codetermination actors are at the center of participation in negotiations on digitalization in the labor process (Haipeter and Schilling, 2023). First of all, it can be generally stated that codetermination represents a “regulatory environment” for the implementation of digital technologies, insofar as the negotiations between the collective actors of management and employee representatives in the company enable certain forms of use of the technologies and limit others (Krzywdzinski et al., 2022). This has been empirically demonstrated not least with respect to wearables and digital assistance systems, which were introduced in the logistic sector and in the production areas of the manufacturing sector. In this case, works councils have proven to be able to negotiate restrictions on data-based performance control, based on their codetermination rights and accepting rationalization effects as the baseline of compromise (Falkenberg, 2021; Krzywdzinski et al., 2022).

However, this finding still says little about concrete strategies and choices works councils have developed in dealing with digitalization. Most studies find that works councils deal with digitization projects in a mostly reactive manner. Reactive means that works councils primarily develop protection claims and try to reduce or compensate for the negative consequences digitalization may have for employment and working conditions. These patterns of action can be distinguished from more active attempts to gain influence on the design of technology and the associated work organization, an approach that seems to be pursued much less frequently.

As Kuhlmann and Voskamp (2019) show in their study on digitalization in mechanical engineering, company representatives tend to be unsettled and overwhelmed, especially in SMEs, due to a lack of resources, limited technical competences and a lack of involvement by management. The situation may be different in larger companies where resources are better and management is more cooperative. Accordingly, the authors contrast strategies of works councils with the attitude of waiting and retreating to consolidated positions of action on the one hand, and claims of proactive participation on the other hand, in the context of which the attempt is made to exert influence on projects about work and organization.

In their study on conflicts over digitization in companies, Rüb et al. (2021) emphasize that the claim of actively influencing digitization processes and developing one's own strategic claims can at best be pursued by resource-rich works councils in large companies, while in smaller companies' resource bottlenecks of the works councils with regard to time, personnel, knowledge and assertiveness make it difficult to help shape the change. Therefore, a reactive protection policy remains a central and for many works councils the only strategy for dealing with digitalization, especially as a competitive discourse dominates in many companies and is also accepted by the works councils, which classifies digital technologies and the associated rationalization and productivity potentials as an unavoidable precondition for competitiveness as well as maintaining locations and employment.

This assessment is shared by Bahnmüller et al. (2023) in their recent analysis of digitization-intensive companies in the metal industry. The authors note that works council action in digitalization processes is generally reactive and aimed at monitoring. Active support for digitization projects is just as uncommon as participation of works councils in teams which are planning and developing digitalization. On the one hand, this is due to resource bottlenecks of the works councils, which do not allow for more extensive activities, but on the other hand also to the assessment that in this way the employees' interests can be represented quite effectively, especially by negotiating employment effects and performance controls.

These findings are in line with the results of the survey conducted as part of the IG Metall—the German metalworkers' union “transformation atlas” (Gerst, 2020). According to this survey, only a smaller proportion of works councils is informed about and involved in change projects at an early stage. From a trade union perspective, Gerst assesses this mode of interest representation by the works councils as “disastrous,” because from his point of view only through more active involvement can employment security and good working conditions be influenced in the longer term in the interests of the employees.

However, there are examples of works councils taking a more active role in shaping digitalization. According to Rego (2022), the prerequisites for this are both a high strategic importance of digitalization as a field of action for the works councils and a strong resource position of the works councils. Under these conditions, the works councils can develop a more active stance on digitalization and develop strategies and claims against company management. There are two conditions in particular that are considered important for works councils to strategically shape digitalization: on the one hand to organize their own work effectively based on clustering competencies in thematic committees, on the other hand to organize direct employee participation within representative works councils' codetermination as a resource for mobilizing the competencies of the workers as experts of their work.

These findings are in line with the analysis of capabilities by Lévesque and Murray (2010), who stress two aspects of capabilities to participate by trade unions or works councils. The first aspect is the internal reorganization of works council work by restructuring bodies and committees, setting up project and working groups, ensuring the internal knowledge acquisition of workers' representatives through training, bringing in external expertise through specialists or also by strategically planning the composition of the works council body from the different specialist areas of a company (see also Niewerth and Massolle, 2022). The second and complementary aspect is the participation of employees. This is basically about using their expert knowledge and at the same time increasing the legitimacy of the representation of interests (Bella et al., 2022; Niewerth and Massolle, 2022). However, these practices seem to be little practiced beyond the boundaries of particularly active works councils' committees. According to Bahnmüller et al. (2023), works councils support forms of management participation within the framework of lean concepts, but do not practice employee involvement as a systematic element of their own work.

In addition, there is a third aspect that is rarely considered in the study of interest representation which is important in the German case, the division of labor between works councils and trade unions as an important basis for the ability of works councils to act. This division of labor is traditionally characterized by mutual support services: trade unions qualify works councils, help them with specific requests, lend them organizational power and relieve them by concluding collective agreements, while conversely works councils monitor compliance with collective agreements, regulate company- and workplace-related issues and recruit members for the trade unions. In this pattern of division of labor, the competences of the trade unions were only called upon by the works councils when needed, an approach that, according to trade union assessments, is no longer sustainable and should be replaced by a more active positioning of the trade unions in order to create the basis for a broader claim of the trade unions to shape the future (Gerst, 2020).

German trade unions have focused these activities in projects in which they try to strengthen the capabilities of the works councils to play a more active role in negotiations and to develop strategies of their own as alternatives to management strategies. In this context several projects have been implemented by the metalworkers' trade union IG Metall which have tried to enable works councils and especially those works councils from SMEs with little resources and capabilities to participate in digitalization issues more actively, to negotiate agreements on how to deal with digitalization projects and to develop own concepts of business strategies based on digital technologies, the most important of them the project “Arbeit und Innovation” together with the Learning Factory of the Chair of Production Systems of Ruhr-University of Bochum (work and innovation) and “Arbeit 2020” (*work 2020*). In the latter project, works councils have been supported by external consultants and trade union officials by up to ten workshops in each case which took place on establishment levels (Haipeter, 2019b). These workshops tried to realize three different goals: Firstly, to develop a digitalization map of the establishment together with employees, secondly, to discuss the political implications of these findings and to identify core topics like employment protection, problems of qualification, deteriorations of working conditions or management problems; and, thirdly, to negotiate these issues with management, trying to pave the way for an agreement which strengthens the opportunities of works councils to participate in digitalization projects and to bring in their own concepts and social aspects. In total, nearly 100 companies and works councils attended in the project, and around 20 agreements have been concluded between works councils and management which focused mainly on procedural rights for the works councils to participate in digitalization projects.

Projects like “work 2020” show that trade unions can give important stimuli to activate works councils mainly from smaller companies to develop new competencies and capabilities to deal more strategically with digitalization issues and to develop a more active approaches of participation. Moreover, they show that negotiated participation in the case of digitalization is less about substantial norms and more about procedural rights of works councils and about opportunities to attend and influence processes of innovation. At the same time, this means that participation in digitalization issues, if it takes place at all, is a continuous task for works councils which requires capabilities of their own in terms of reorganizing the work within works councils' committees (see also Rego et al., 2021). This is a core precondition in terms of agency for the institution of codetermination to shape digitalization and the introduction of AI in firms.

## AI implementation—challenges for works councils

Against the background of the theoretical key concepts and actual empirical findings on the importance of labor relations and especially of labor politics of workers' representatives in the context of the introduction of digital technologies, the following chapter gives a deeper insight how works councils deal or are able to deal with the introduction of AI. Besides the shift to remote working in the wake of the COVID-19 pandemic (Kötter et al., 2023), the introduction of AI is certainly one of the biggest current challenges for works councils, as AI systems could lead to substantial changes in work processes and new qualification requirements for employees. In general, the implementation of AI can have a direct impact on employees and their activities (human-centered) or primarily on technical processes and thus only secondarily on employees (technology-centered) (Huchler, 2023; Pfeiffer, 2023). Taking these possible different paths in account, the introduction of AI in the company confronts works councils with vital challenges. The first question that arises are the competences necessary for understanding and dealing with AI, as well as anticipating the far-reaching changes that the introduction of AI can mean for work processes.

A clear stance is needed that pushes for the enforcement of co-determination rights regarding AI. Often employee representatives are overwhelmed in the first step and realize that there is no suitable set of rules for such a case. On the part of the employees, the committees might be confronted with reservations and fears, even though there are not yet reliable figures on the long-term effects of AI on employment (Ver.di, 2020). A comprehensive stakeholder sensitization is needed, which includes in particular a technology impact assessment.

In addition, as in the case of the AI implementation at the German company Siemens which will be analyzed later in this article, AI applications are often not readily recognizable and are mixed with automation and general digitalization processes (Grasy and Seibold, 2023). The fact that a generally applicable and comprehensive definition is often still lacking (Höfers and Schröder, 2022) rises points of conflict where the employer side restricted the concept of AI to self-learning systems alone and thus wanted to undermine the right of the works council to have a say (Grasy and Seibold, 2023). However, since the amendment Works Council Constitution Act in 2021 (BetrVG 80, 3) allows the co-determination body to call in experts to advise it.

Taking this legal base of AI implementation in account, international comparative studies underline, that in German cases of AI implementation is a tendency toward social partnership solutions, which often take a similar path (Doellgast and Kämpf, in press). AI is often seen as a “cross-cutting issue” with effects on areas of employment and labor conditions as well as collective bargaining policy. A particular argument here is the reference to the EU AI Act, which also addresses the ethical basis of “AI made in the EU” and excludes certain types of AI (high risk) from the outset. At the same time, AI systems are often still a “black box”—whether personal data can be collected, for example, can often only be examined after purchase (Grasy and Seibold, 2023). Following theses authors, co-determination must become a direct part of the introduction process.

At the same time, the introduction of AI can offer an opportunity to enter into negotiations, e.g., to force further training and retraining, but it can also lead to more stress and anxiety (Doellgast, 2022). Trade unions (and in the German case, first of all works councils) are confronted with three main problems: the threat of job losses, special requirements for data protection and the challenge of organizing outsourced employees, e.g., in subcontractors. Europe and Germany have comparatively strong regulations with regard to data protection. Solutions to these problems can be attempts to influence government legislation; negotiating new labor standards through trade unions; and at plant level company agreements. This level and the challenges and approaches associated with it will be examined further in the following example of Deutsche Telekom.

## The qualitative case analysis—process of developing a works agreement for artificial intelligence systems

In the context of a qualitative analysis the question is explored of how works councils can have a say before the introduction of new AI systems already begins. Since valid works agreements on IT are no longer sufficient when AI is already introduced, the core criteria for a model company agreement on AI are being worked out during this analysis. In order to understand the contextual conditions and the participation of works councils in the introduction of AI solutions at Deutsche Telekom, a comprehensive document analysis of company agreements and open guideline interviews with members of the group works council (GWC) were used. The aim was to draw on the experiential knowledge of workers' representatives to enable a reconstruction of the decision-making process. Deutsche Telekom was also a project partner in the BMAS-funded project “humAIn work.lab,” which investigated risks and opportunities in the application of AI at work (in the period from 2020–2023). The underlying transfer research concept enables the work-oriented implementation of research projects with a focus on the transfer of knowledge between scientific disciplines and practitioners. This knowledge transfer as a constitutive component of the research process contributes significantly to an interlocking of research and social practice (Schäfer et al., 2022, p. 129–132). In general, this method provides “exclusive insights into the complexity of structural contexts and processes of change in systems of action, such as decision-making structures and problem-solving in organizations and institutions” (Liebold and Trinczek, 2009, p. 53). To be able to track the work steps of a works council committee in this context, a works council committee was to be accompanied at intervals of several weeks over a period of 2 years. In the course of intensive cooperation (Schäfer et al., 2022), with the group works council of Deutsche Telekom Service GmbH, the data collected in advance was condensed during the field analysis through the perspective of active works councils. The dialogic interviews with works council members of Deutsche Telekom Service GmbH are recorded in detailed protocols and supplementary visual material and evaluated in several phases. The analysis of the collected data is aimed at identifying core criteria that facilitate the development of company agreements on AI.

## Case study Deutsche Telekom

### Operating agreement for artificial intelligence systems

The strategy of Deutsche Telekom's group works council (GWC) was chosen as a case study for two reasons: because this company develops Artificial Intelligence (AI) based tools which makes it a vanguard company of the German IT sectors and, on the other hand, because its works council plays a very active role in the regulating and shaping the AI introduction processes within the company (Doellgast and Kämpf, in press).

The company offers products and services in the areas of fixed network, mobile telephony, Internet and Internet TV for private customers as well as information and communication technology solutions for major and business customers. The former public company Deutsche Telekom was privatized in 1996, and in 2022 considered the largest telecommunications company in Europe. The German government still holds nearly 32% of the company's stock in 2022 and counts with 220,000 employees worldwide and more than 90,000 of them in German locations. Because of its history as a former public company, the Deutsche Telekom AG is still highly unionized by nearly 80%, despite being a high-tech company.

Codetermination at the Deutsche Telekom takes place in the form of a multilevel system, composed of local works councils, central works councils for the divisions and subsidiaries of the company and the group works council, which is composed of members of the different central works councils. Issues related to the implementation of IT systems are dealt with in the group works councils as many IT systems are used in the whole group and not only in certain divisions or subsidiaries of the corporation. In total, the group works council (GWC) consists of 27 members from 10 delegate areas. The GWC has established a special committee dealing with IT issues, the IT committee, which is composed of 4 GWC members and other works councils from the central works councils and from local works councils which are at the same time experts in dealing with IT issues (Bargmann, 2022).^3^

A core approach toward AI developed in the GWC of Deutsche Telekom is that AI is not to be regarded as a finished technology, but as a learning system of information technology. In this view, AI evolves to perform tasks, optimizes itself and solves problems by independently recognizing patterns, drawing conclusions and preparing or making decisions. Taking these patterns into account, the introduction and the use of AI is an ongoing process of a deep technological transformation.

### The AI Manifesto

In this context, in October 2022 the so called “AI Manifesto” was concluded between the Deutsche Telekom management and the group works council. The “AI Manifesto” is an agreement between the GWC and the company management about the introduction and implementation of AI within all the section of the company. Apart from regulating AI implementation, the Manifesto at the same time can be regarded as a new type of agreement between works councils and management because it represents a new forms of agile company agreement.^4^

Basically, the agreement refers to national and international (EU) legal regulations and (technical) standards and supplements the Group Works Agreement on IT Systems, the Group's Digital Ethics Guidelines for dealing with artificial intelligence and General Data Protection Regulation (DGPR). Basic positions were laid down also referring to the latest legal amendment of the German Work Constitution Act from July 2021 that stipulates that employees have to be informed about possible interactions with learning machines, that personnel-relevant decisions must not be made by AI or that AI is not allowed to be used for surveillance. Based on this, common goals and procedures were agreed upon concerning on the introduction and use of artificial intelligence the generally applicable regulatory framework, quality requirements, dealing with risks or the introduction of a group of experts composed of management and works councils. Another important point of the agreement is that it includes the rule that employees of Deutsche Telekom have to be at the center of all operational decision-making process concerning AI. In detail, the main principles of the Manifesto are the following:

First, the interaction between employees and learning machines has to be designed in such a way that employees are informed about the fact that they are interacting with such a machine. In line with the already existing agreements on IT Systems, the Manifesto says that employees have to be protected against machine control of performance and behavior and prohibit the use of unauthorized humane data. Only human decision makers are attributed the right to draw conclusions relevant to human resources that could have legal effects on employees or significantly influence them in a similar way. Employees who are indirectly affected by machine conclusions with personal effects can request a review of the system decision from those responsible. Furthermore, according to the agreement AI systems will not be used to analyze, influence or control employees' emotions or mental state. Employee biometric data and AI systems designed to improve employee wellbeing will only be used if permitted by other company agreements.

Besides these more basic rules, the *AI Manifesto* includes procedural rules about how to cope with the implementation of AI systems.

At first management and the group works council agreed on quality, trust factors, and quality checks of AI in which also works council members are involved: Legal and regulatory compliance of AI solutions, transparency, compatibility with the Digital Ethics “AI Guidelines,” usefulness in the performance process, risk appropriateness, controllability, protection of personal rights, ergonomics, social compatibility, good work, robustness, and sustainability.

Secondly, the agreements stipulate that the group works councils should be informed in early stage about the data sources of the AI system and assessments of the informative value and integrity of the data system description, model of the AI system, plans for evaluating the model quality in ongoing operation and emergency concept, depending on the respective risk classification and planning phase. Works councils can demand unscheduled monitoring from those responsible for the system if there are indications that the system is not being used in accordance with this agreement.

Thirdly, the agreement states that a joint AI-expert group with the management (4 members) has to be implemented. This group receives, together with the GWC, the information on the results and methods of system training and testing. The expert group is continuously involved in the development of impact assessment procedures, standards for the assessment of risk dimensions and their probabilities of occurrence. Apart from this and depending on special issues, group works council is allowed to call in further experts according to § 80.3 Work Constitution Act to create generalizable procedures for co-determination and quality assurance on AI systems. Finally, the operational functional managers for the AI applications and representatives of the works councils (IT Committee) will be continuously qualified to put into practice this Manifesto (Höfers and Schröder, 2022).^5^

### The AI Manifesto in practice

**Figure 1:**
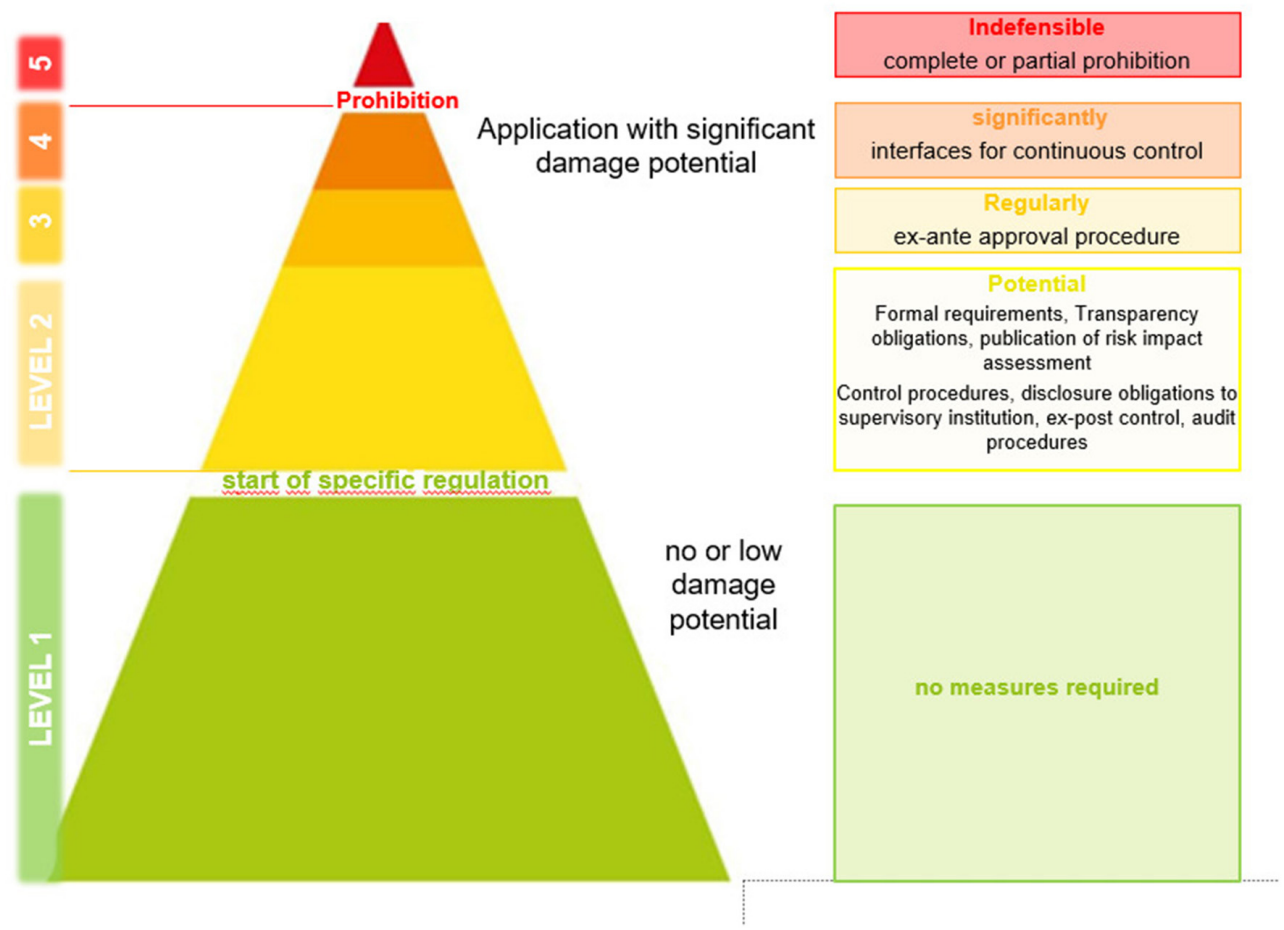
Criticality levels of AI introduction. Source: Deutsche Telekom.

In this context the group works council has developed a pyramid of criticality levels of AI based applications and systems, which refers mainly to the AI-Strategy of the current Federal Government and determines the damage potential of an AI and provides for measures and actions accordingly (see Figure 1).^6^ Depending on the risk classification of planned AI applications, different possible actions for the expert group are defined. The potential for harm of the application is assessed in five levels. Level 1 (green) refers to the introduction of AI with no or little potential for harm to employees in the sense that it does not interfere with personal basic rights. In this case, no separate regulatory measures in company agreements are required on the part of the works council. Level 2 (yellow) describes a certain potential for harm by reducing the decision-making autonomy of the employee through digital twins. In this case, management is obliged to comply with certain transparency obligations and to carry out a risk impact assessment. This includes specific control and evaluation procedures. Levels 3 and 4 (orange) indicate AI applications with regular and significant potential for harm by the potential use of sensor technology that detects and processes employee behavior. These are either reviewed through ex-ante approval procedures or prohibited if necessary. Level 5 (red) indicates an area of AI application that is considered unacceptable and that is rejected by the works council. These AI applications would have the potential to monitor the employee's behavior or performance with corresponding consequences for pay development (which is forbidden par § 87.6 BetrVG). If these technical possibilities can be ruled out through an evaluation, the group works council can partially agree to this AI application afterwards. In essence, level 5 covers with all the regulatory areas of section 87 (1) 6 and 10 of the Works Constitution Act, which are subject to the co-determination of the works council. In this case management is not allowed to introduce this AI without its consent.

The criticality levels marked in Figure 1 are the first step to an operationalization of the programmatic statements in the Manifesto. In this way, the management of Telekom and the GWC have developed an ethical framework that will enable them to introduce AI systems in a dialogue-based and structured manner (Höfers and Schröder, 2022).

**Figure 2:**
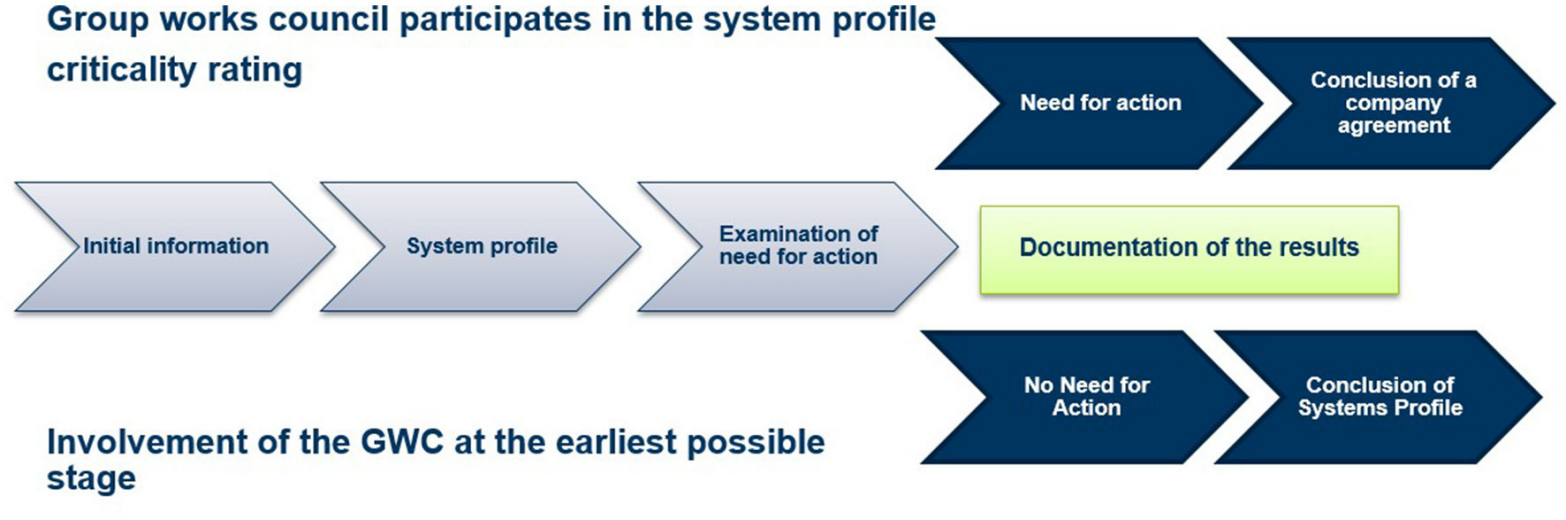
Digital roadmap. Source: Deutsche Telekom.

The second step of operationalization of the AI-Manifesto and the pyramid of critical levels was the development of a so called “digital roadmap” (Doellgast and Kämpf, in press). This roadmap defines steps of participation the GWC can potentially make use of, in line with the review of the rating of the AI. These steps of participation are about renegotiating existing agreements on IT systems based on the results of the assessments made; given this, the digital roadmap can be regarded as “learning” regulation which allows to adapt regulations to new facts. In practice this means that after the initial information by the management has taken place, the GWC participates in the development of so-called system profiles, which form the basis for both a review of the system and a possible need for action by the works council. If, after documenting the audit results, it is determined that there is no need for action (usually at criticality level 1), the profile is closed and the existing IT company agreement should not be renegotiated. If, however, a need for action is identified after the audit (usually at criticality level 2), elements of the IT company agreement have to be renegotiated (see Figure 2).

Therefore, the digital roadmap presents the base for third step of AI introduction by Deutsche Telekom, the development of new forms of agile company agreements on IT systems. These agile agreements can be regarded as a strategic change toward a digitalisation of co-determination processes. At its core is a profile procedure for IT systems. Linked to the Manifesto programmatic, it is controlled by a project management software JIRA@BR (Bargmann, 2022) which is based on a new version of the GWC agreement on the planning, introduction, use and modification of IT Systems (GWCA IT Systems) from March 2021 and on the GWC on Digital Cooperation (GWCA DC).

While so far, the GWC used to prepare separate, specific company agreements for each new digital tool, now on the base of the digital roadmap the works councils are able to develop new and comprehensive company agreements that sets labor standards. In this context the GWC members have recognized that the preparation of independent from each other and isolated company agreements on continuous technological innovation is too time consuming, especially in view of the rapid development of AI. New agile company agreements include individual rules which always apply, while other sections are to be understood as core principles which should always be taken into account in the context of technical innovation processes. This refers mainly to the content of § 87 Works Constitution Act and the protection of the basic personal rights of employees. In this context, the rights of co-determination of the works councils are no longer contested in negotiations with the management; they are taken as given by the procedural rules.

At the same time, these forms of accelerated co-determination procedures offer advantages for management, as it allows finally to speed up the introduction of digital technologies in general and AI in concrete terms. When new AI systems are introduced, the following process of labor policy applies: First, initial information of the GWC by the management at the earliest possible opportunity; second, draw up a profile of the program; and third, check need for action referring to the question if the basic rights of the employees are met. The AI implementation is thereby examined by the GWC from an application perspective.

**Figure 3:**
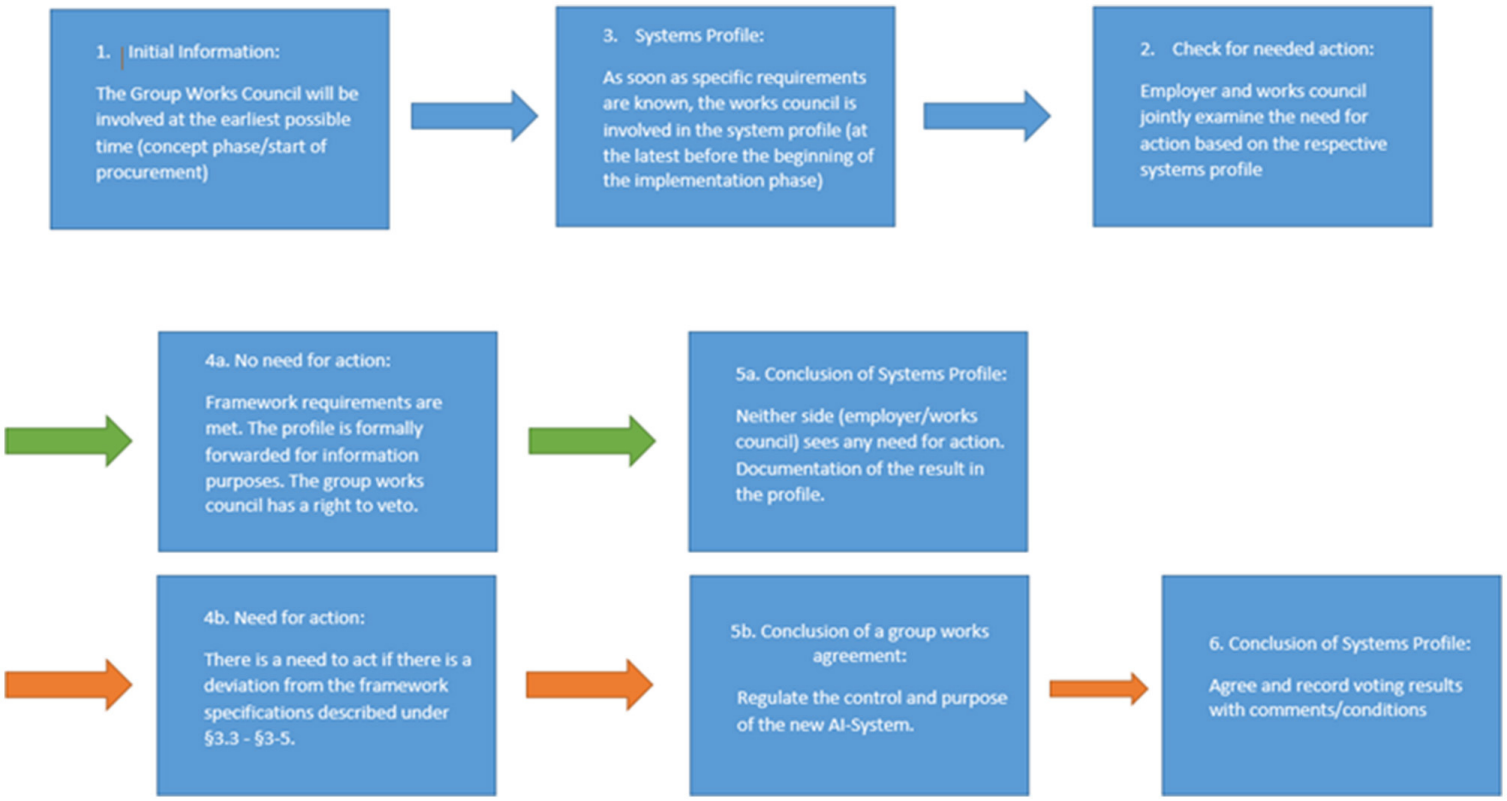
Operational practice: IT co-determination process including AI. Source: Deutsche Telekom, own preparation.

GWC members reported, that veto rights until today have rarely to be used—often rather in the case of misunderstandings. However active control of the process is still important in the opinion of the workers' representatives. The result is finally an agile “dual model” of IT co-determination. It is characterized firstly by a general, fundamental and overarching set of rules applicable to all IT systems (GCA IT systems), which are and no longer negotiated, and secondly the concentration in the day-to-day business of ongoing co-determination on those IT systems that require deviations from these core principles. The procedural core of this is the so-called system profile:

Figure 3 illustrates this agile model of co-determination concerning the introduction of IT-systems established by the AI Manifesto. The works council has to be involved from the beginning of the introduction process. An important role is played by the technology assessment of AI (system profile). Possible rationalization processes resulting from the use of AI are also dealt with proactively, because management has to present the (planned) digitalization goals in advance. The works council is informed in this regard and then can become active itself. This is particularly important as the system automatically assigns enough work so that a possible reduction in the workload of individual employees cannot be identified easily. Moreover, sometimes it is not even clear which tasks are omitted or have already been taken over by AI. In order to cope with these sophisticated problems, works council members receive continuous trainings. The costs of these are fully covered by the employer under section 37.6 of the German Works Constitution Act. There is also a regular exchange with the employee representatives on the supervisory board (Höfers and Schröder, 2022).

These three steps of participation in the context of AI-introduction underline a strategic re-orientation of co-determination on AI issues in the Deutsche Telekom AG. It presents new and innovative approaches of agile procedural rules for co-determination of works councils. However, implementation of the new regulations 1 year after the conclusion of the Manifesto are still in a learning phase and there is still a need for further empirical analysis of how these agreements work in practice.

### Contextualization of the case study

The qualitative analysis has described the way in which the group works council at Telekom has reached a social partnership agreement in the context of AI implementation. But companies of the telecommunication sector play a special role here (Doellgast, 2022), so the following chapter refers to two other actual examples with their approaches to the introduction of AI to finally contextualize the Telekom case.

The first example is Siemens, the largest industrial manufacturing company in Europe, specialized in industrial automation and industrial software. Siemens is already developing and using AI itself (e.g., in personnel processing; as a supporting and relieving chatbot), but in comparison with Deutsche Telekom still has no fundamental company agreement on AI with regard to co-determination (Grasy and Seibold, 2023). Until 2023 only preliminary work has been done by general works council. All relevant functions and forms of use of the respective AI applications have to be presented in profiles, so called “AI cards.” The applications and its tasks as well as the possible consequences on employment and labor conditions are to be made comprehensible and clear in this way and help to reduce uncertainty among the employees.

Although the effects of new applications on employees and the resulting measures can be grasped in this way, the general works council is only acting after the introduction of AI reactively until today. At the same time, the group works council has been able to establish guidelines about data protection and data storage and, more broadly, basic ethical considerations that are recognized by the Siemens management. For definitional standards, however, the committee is placing more expectations in definitions from the EU. The group works council was accompanied in this process by an expert team of the German metal union, IG Metall. At this stage of development, Siemens is still relying on “weak” AI, i.e., rather AI assistance, which in forms of chatbots is so far only intended to relieve employees internally. Nevertheless, this could also be a step toward job cuts, as the first contact with customers could also be taken over by a bot (Doellgast, 2022).

The second example is International Business Machines Corporation (IBM), Germany. This world leading company of IT-services is already one step ahead of Siemens. IBM has reached a company agreement on AI since 2020. Similar to the case of Deutsche Telekom, they group works council and the management have developed a framework agreement on the conditions for the introduction and operation of IT systems, which explicitly excluded work performance and behavioral control of the employees. This company agreement later became the basis for the group agreement especially on AI tools—a process which, according to Doellgast et al. (2022), is relatively known in the German system of labor relations. At the same time, internal ethics guidelines also existed in advance. The EU Ethical Principles for Trustworthy AI and the study by the Bundestag's Enquete Commission on the Potential of AI were also consulted.

Like in the Telekom case, also at the IBM group works council there was ultimately great interest to reach a company agreement, which took place in an open and solution-oriented process with the management. Representatives of the group works council and the representatives of the severely disabled employees, together with HR staff and in-house IT specialists of IBM, were able to learn about the technical basis of AI in a joint series of workshops and at the same time collect topics for a possible company agreement. The focus was on the primacy of human decision-making and the possibilities for intervention as well as exclusion of social discrimination. Like in the case of Deutsche Telekom, damage categories or risk clusters were established here, which demonstrates a certain way of dealing with the respective application of AI. Representatives of IBM's works council have announced that it has been one of the first large companies in Germany which has established a company agreement on AI. Analogous to Siemens, IBM also works with “AI fact sheets” and has similar to Deutsche Telekom—an ethics council that examines new AI applications. Nevertheless, the definition of AI and the question of when it is an intelligent system has not yet been comprehensively clarified in the case of IBM (Remers, 2023).

The examples of Siemens and IBM Germany also underline some general results of the qualitative analysis on the Telekom case. Ethic frameworks like the AI- Manifesto and instruments and methods like the digital roadmap seem to be able to support the development of new types of agile company agreements in the context the introduction of AI solutions. Looking at the broader landscape of German labor relations and codetermination and the opportunities to learn from the examples of these large companies, it should be reflected that the power resources of workers' representatives to exert influence in the development of AI-projects in these companies are much greater than they are in the procurement of AI-solutions from external providers or from small start-ups which develop AI solutions. Therefore, the qualitative research results have strong links with the concept of the path dependency of companies like the Deutsche Telekom that still presents high union organizing power and a strong works council with a multi-level system. For external providers of AI-solution the results concerning workers' participation on AI-introduction may look quite different, where research has lot of to undertake in the near future.

## Summary and outlook

The contextualization of the results of the qualitative case study on Deutsche Telekom underlines the importance of participation and the power resources of the respective actors as well as the role of negotiations and conflicts in the labor process and the relevance of the production and business models these are embedded in. These are key factors that help to explain AI implementation both in terms of the development of single company cases and in terms of the differences between cases. Given this, the analyzed Telekom case underlines the importance of the concept of production models. Large companies with a unionized workforce and an established multi-level system of works councils are able to offer favorable conditions for institutionalized workers' participation. In the case of the Deutsche Telekom, this condition overlapped with a tradition of social partnership that characterized labor relations and therefore conflicts in the labor process in a former public company. Based on these social relationship, management and the group works council developed new agile forms of work organization and participation to strengthen high-tech market strategies in a tough competitive environment.

At the same time, the case study underlines the importance of participation by works councils in the context of the introduction digital technologies and AI. At the Deutsche Telekom, the group works council has succeeded to develop and agree new and agile forms of participation with management as an innovative answer to AI challenges, based both on institutional power resources and the relations of social partnership with the management. This agile approach could also include a transformation process of the works council itself and a need for specific and agile-compatible qualifications of its members (Niewerth and Massolle, 2022).

Finally, in line with the concept of labor process, the Telekom case and the examples of Siemens and IBM Germany show that in ongoing technological and organizational transformation processes permanent negotiations between management and employee representatives are needed to implement agreements that adapt to deeply changing situations in employment issues. The qualitative empirical analysis has shown that corporate agreements like the “AI Manifesto” and the “digital roadmap” are able to open a road to a consensus between management and works councils to find a common way to deal with the digital transformation process of AI implementation. On the one hand, these negotiations go along also with a professionalization process of the works councils to cope with technological and organizational issues on the central level of the GWC. This centralization of qualification, competencies and capabilities to act might, on the other hand, produce a challenge within the multi-level system of employee participation to communicate such compromises of workplace democracy (Dukes and Streeck, 2023) from the central company level to the nearly one thousand works councils members on local level within the company and to advertise the political legitimacy of these labor compromises. But finally more in-depth empirical analyses are needed on the critical functioning of these company agreements on AI in the further course of time.

## Data availability statement

The original contributions presented in the study are included in the article/supplementary material, further inquiries can be directed to the corresponding author.

## Author contributions

TH: Writing – original draft. MW: Writing – original draft. J-TD: Conceptualization, Supervision, Writing – original draft. SS: Methodology, Writing – original draft, Writing – review & editing.

## Footnotes

^1^In the multi-level system of co-determination (local works council, group works council, European works council), the group works council at Telekom group level leads the negotiations with management on the introduction of AI.

^2^See: https://www.europarl.europa.eu/news/en/headlines/society/20230601STO93804/eu-ai-act-first-regulation-on-artificial-intelligence?, https://www.ki-strategie-deutschland.de/home.html (06-26-2023).

^3^See; Deutsche Telekom (2023): HR Factbook 2022, Menschen. Fakten. Entwicklungen.

^4^See: https://www.telekom.com/de/konzern/details/telekom-verpflichtet-sich-auf-ki-ethik-1025794 (15. 10. 2023).

^5^Source: Manifesto between the Deutsche Telekom Group and the Group Works Council on the introduction and use of information technology systems, October 21, 2022.

^6^See: https://www.ki-strategie-deutschland.de/home.html.
